# Crystal structure of tris­(*trans*-1,2-cyclo­hexa­ne­diamine-κ^2^ *N*,*N*′)chromium(III) tetra­chlorido­zincate chloride trihydrate from synchrotron data

**DOI:** 10.1107/S2056989016005788

**Published:** 2016-04-12

**Authors:** Dohyun Moon, Jong-Ha Choi

**Affiliations:** a Pohang Accelerator Laboratory, POSTECH, Pohang 37673, Republic of Korea; b Department of Chemistry, Andong National University, Andong 36729, Republic of Korea

**Keywords:** crystal structure, 1,2-cyclo­hexa­nedi­amine, tetra­chlorido­zincate chloride double salt, chromium(III) complex, hydrogen bonding, synchrotron radiation

## Abstract

The structure of the title double salt, [Cr(*rac*-chxn)_3_][ZnCl_4_]Cl·3H_2_O (chxn is *trans*-1,2-cyclo­hexa­nedi­amine; C_6_H_14_N_2_), has been determined from synchrotron data. The Cr^III^ ion is coordinated by six N atoms of three chelating chxn ligands, displaying a slightly distorted octa­hedral coordination environment. The distorted tetra­hedral [ZnCl_4_]^2−^ anion, the isolated Cl^−^ anion and three lattice water mol­ecules remain outside the coordination sphere. The Cr—N(chxn) bond lengths are in a narrow range between 2.0737 (12) and 2.0928 (12) Å; the mean N—Cr—N bite angle is 82.1 (4)°. The crystal packing is stabilized by hydrogen-bonding inter­actions between the amino groups of the chxn ligands and the water mol­ecules as donor groups, and O atoms of the water mol­ecules, chloride anions and Cl atoms of the [ZnCl_4_]^2−^ anions as acceptor groups, leading to the formation of a three-dimensional network. The [ZnCl_4_]^2−^ anion is disordered over two sets of sites with an occupancy ratio of 0.94:0.06.

## Abstract 2

The Cr^III^ ion in the title compound is coordinated by six N atoms of three chelating 1,2-cyclo­hexa­nedi­amine (chxn) ligands, displaying a distorted octa­hedral environment. The crystal packing is stabilized by extensive hydrogen-bonding inter­actions between the N—H groups of the chxn ligands, O—H groups or O atoms of the water mol­ecules, chloride ions and Cl atoms of the disordered [ZnCl_4_]^2−^ anions.

## Chemical context   

*trans*-1,2-Cyclo­hexa­nedi­amine (chxn) can coordinate to a central metal ion as a bidentate ligand *via* the two nitro­gen atoms, forming a five-membered chelate ring. The synthetic procedures, crystal structures and detailed spectroscopic properties of such [Cr(chxn)_3_]^3+^ complexes with chloride or nitrate anions have been reported previously (Morooka *et al.*, 1992 ▸; Choi, 1994 ▸; Kalf *et al.*, 2002 ▸). Since counter-anionic species play a very important role in coordination chemistry and supra­molecular chemistry (Fabbrizzi & Poggi, 2013 ▸; Santos-Figueroa *et al.*, 2013 ▸), changing the type of anion can also result in different structural properties. With respect to the tetra­chlorido­zincate anion, [ZnCl_4_]^2−^, the crystal structures of complexes with trivalent chromium have been determined for [Cr(NH_3_)_6_][ZnCl_4_]Cl (Clegg, 1976 ▸), [Cr(en)_3_][ZnCl_4_]Cl (en is ethyl­enedi­amine; Pons *et al.*, 1988 ▸) and *trans-*[Cr(NH_3_)_2_(cyclam)][ZnCl_4_]Cl·H_2_O (cyclam is 1,4,8,11-tetra­aza­cyclo­tetra­decane; Moon & Choi, 2016 ▸). However, a combination of the [Cr(chxn)_3_]^3+^ cation with [ZnCl_4_]^2−^ and Cl^−^ as anions is unreported. In order to confirm that the resulting structure belongs to a double salt with [ZnCl_4_]^2−^ and Cl^−^ anions and does not contain a [ZnCl_5_]^3−^ anion, we prepared this material and report here on the mol­ecular and crystal structure of [Cr(*rac*-chxn)_3_][ZnCl_4_]Cl·3H_2_O, (I).
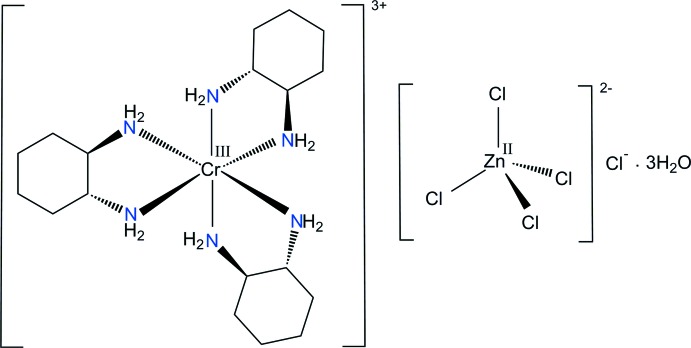


## Structural commentary   

First of all we performed a single-crystal structure analysis of the starting complex [Cr(chxn)_3_]Cl_3_·2H_2_O with 98 K synchrotron data to determine the exact composition and coordination geometry of the Cr^III^ ion. The complex crystallizes in the space group *I* 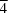
2*d* with eight formula units in a cell of dimensions *a* = 18.893 (3) and *c* = 14.069 (3) Å. The Cr—N(chxn) bond lengths are in the range 2.0723 (19) to 2.0937 (19) Å, and the N—Cr—N bite angles are in the range 82.53 (7) to 82.69 (10)°. In comparison with the bond lengths and angles of the structure of this complex determined with 223 K data (Kalf *et al.*, 2002 ▸), there are no remarkable differences, and also the the crystal packing has virtually identical features.

**Figure 1:**
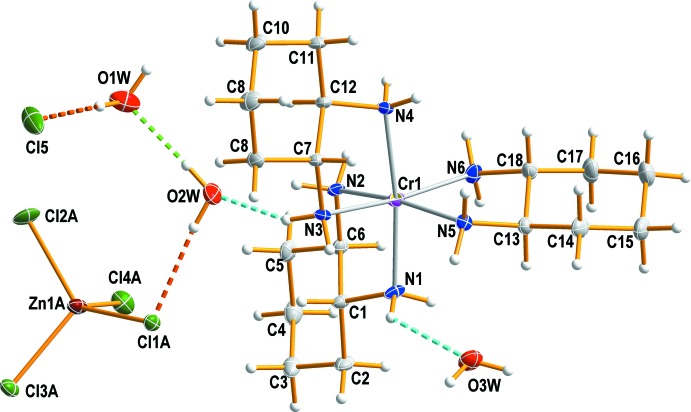
The structures of the mol­ecular components of the title double salt, drawn with displacement parameters at the 50% probability level. Dashed lines represent hydrogen-bonding inter­actions.

Fig. 1 ▸ shows the mol­ecular components of the title compound, (I), which consists of a discrete complex cation [Cr(*rac*-chxn)_3_]^3+^, three lattice water mol­ecules, together with one tetra­hedral [ZnCl_4_]^2−^ and one isolated Cl^−^ counter-ion. The nitro­gen atoms of the three 1,2-cyclo­hexa­nedi­amine ligands define a distorted octa­hedral coordination environment around the Cr(III) ion with a mean N—Cr—N bite angle of 82.1 (4)°. The resulting five-membered chelate rings of chxn ligands have the expected stable *gauche* conformation. The Cr—N(chxn) bond lengths are in the range 2.0737 (12) to 2.0928 (12) Å, in good agreement with those determined in [Cr(*RR*-chxn)_3_](NO_3_)_3_·3H_2_O (Morooka *et al.*, 1992 ▸) and [Cr(*rac*-chxn)_3_]Cl_3_·2H_2_O (Kalf *et al.*, 2002 ▸). The disordered tetra­hedral [ZnCl_4_]^2−^ anion, the isolated Cl^−^ anion and the three water mol­ecules remain outside the coordination sphere of Cr^III^. The complex [ZnCl_4_]^2−^ anion is distorted due to its involvement in hydrogen-bonding inter­actions. The [ZnCl_4_]^2−^ and Cl^−^ anions are well separated by van der Waals contacts and consequently there is no basis for describing the Zn^II^ species as a distorted [ZnCl_5_]^3−^ anion.

## Supra­molecular features   

**Figure 2:**
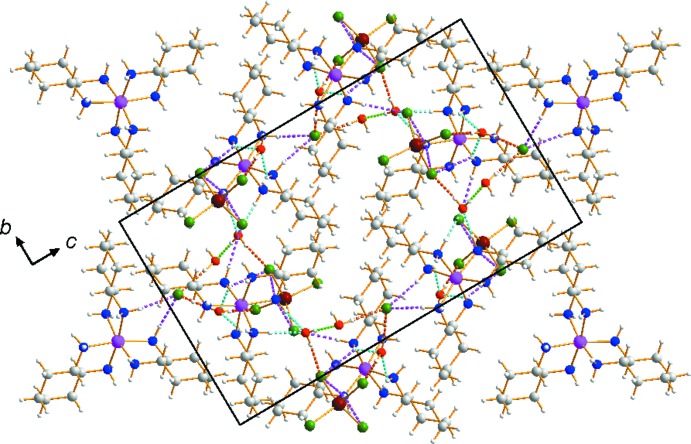
The crystal packing in the title double salt viewed perpendicular to the *bc* plane. Dashed lines represent hydrogen-bonding inter­actions: N—H⋯Cl (pink), N—H⋯O (cyan), O—H⋯O (light green) and O—H⋯Cl (orange). The minor disorder components of the [ZnCl_4_]^2−^ anion have been omitted for clarity.

Extensive hydrogen-bonding inter­actions occur in the crystal structure (Table 1 ▸), involving the N—H groups of the chxn ligands and the O—H groups of the lattice water mol­ecules as donors, and the chloride ions and Cl atoms of the disordered [ZnCl_4_]^2−^ anions and water O atoms as acceptors. The supra­molecular architecture gives rise to a three-dimensional network structure (Fig. 2 ▸).

## Database survey   

A search of the Cambridge Structural Database (Version 5.36, May 2015 with last update; Groom *et al.*, 2016 ▸) shows that there are three previous reports for Cr^III^ complexes containing three chelating chxn ligands, *viz*. [Cr(*RR*-chxn)_3_](NO_3_)_3_·3H_2_O (Morooka *et al.*, 1992 ▸), [Cr(*rac*-chxn)_3_]Cl_3_·2H_2_O (Kalf *et al.*, 2002 ▸), and [Cr(*RR*-chxn)_3_][Co(*SS*-chxn)_3_]Cl_6_·4H_2_O (Kalf *et al.*, 2002 ▸). The structure of any double salt of [Cr(chxn)_3_]^3+^ with an additional [ZnCl_4_]^2−^ anion has not been deposited.

## Synthesis and crystallization   

Commercially available (Aldrich) *racemic trans*-1,2-cyclo­hexa­nedi­amine was used as provided. All other chemicals with the best analytical grade available were used. The starting material, [Cr(*rac*-chxn)_3_]Cl_3_·2H_2_O was prepared according to the literature (Pedersen, 1970 ▸). The crude trichloride salt (0.22 g) was dissolved in 10 mL of 1 *M* HCl at 313 K and 5 mL of 1 *M* HCl containing 0.5 g of solid ZnCl_2_ were added to this solution. The resulting solution was filtered and allowed to stand at room temperature for one week to give block-like yellow crystals of the tetra­chlorido­zincate(II) chloride salt suitable for X-ray structural analysis.

## Refinement   

Crystal data, data collection and structure refinement details are summarized in Table 2 ▸. All H atoms were found from difference maps but were placed in geometrically idealized positions and constrained to ride on their parent atoms, with C—H = 0.99–1.00 Å and N—H = 0.91 Å, and with *U*_iso_(H) values of 1.2 or 1.5*U*_eq_ of the parent atoms. The hydrogen atoms of water mol­ecules were restrained using DFIX and DANG commands during the least-squares refinement (Sheldrick, 2015*b* ▸). The [ZnCl_4_]^2−^ anion was refined as positionally disordered over two sets of sites with a refined occupancy ratio constrained to 0.94:0.06 in the last refinement cycles.

## Supplementary Material

Crystal structure: contains datablock(s) I. DOI: 10.1107/S2056989016005788/wm5284sup1.cif

Structure factors: contains datablock(s) I. DOI: 10.1107/S2056989016005788/wm5284Isup2.hkl

CCDC reference: 1472901

Additional supporting information:  crystallographic information; 3D view; checkCIF report

## Figures and Tables

**Table 1:** Hydrogen-bond geometry (Å, °)

*D*—H⋯*A*	*D*—H	H⋯*A*	*D*⋯*A*	*D*—H⋯*A*
N1—H1*A*⋯Cl5^i^	0.91	2.40	3.2535 (15)	157
N1—H1*B*⋯O3*W*	0.91	2.36	3.0178 (16)	129
N2—H2*A*⋯O2*W*	0.91	2.01	2.9051 (17)	166
N2—H2*B*⋯Cl2*A* ^ii^	0.91	2.45	3.2197 (14)	142
N2—H2*B*⋯Cl3*B* ^ii^	0.91	2.36	3.180 (18)	150
N3—H3*A*⋯O2*W*	0.91	2.13	2.9832 (16)	156
N3—H3*B*⋯Cl1*A* ^iii^	0.91	2.52	3.2574 (13)	138
N3—H3*B*⋯Cl3*A* ^iii^	0.91	2.77	3.4547 (16)	133
N3—H3*B*⋯Cl2*B* ^iii^	0.91	2.67	3.471 (10)	147
N3—H3*B*⋯Cl4*B* ^iii^	0.91	2.68	3.35 (2)	131
N4—H4*A*⋯Cl1*B* ^iv^	0.91	2.74	3.473 (11)	138
N4—H4*B*⋯Cl2*A* ^ii^	0.91	2.64	3.4267 (15)	146
N4—H4*B*⋯O1*W* ^ii^	0.91	2.39	2.9804 (17)	123
N5—H5*A*⋯Cl3*A* ^iv^	0.91	2.51	3.4245 (14)	178
N5—H5*A*⋯Cl4*B* ^iv^	0.91	2.73	3.634 (19)	173
N5—H5*B*⋯Cl1*A* ^iii^	0.91	2.74	3.3664 (16)	127
N5—H5*B*⋯O3*W*	0.91	2.22	2.9724 (17)	140
N6—H6*A*⋯Cl5^i^	0.91	2.39	3.2474 (14)	158
O1*W*—H1*O*1⋯Cl5	0.85 (1)	2.24 (1)	3.0878 (17)	179 (2)
O1*W*—H2*O*1⋯Cl4*A* ^ii^	0.84 (1)	2.28 (1)	3.1170 (13)	174 (2)
O2*W*—H1*O*2⋯Cl1*A*	0.83 (1)	2.28 (1)	3.1140 (12)	175 (2)
O2*W*—H1*O*2⋯Cl2*B*	0.83 (1)	2.45 (1)	3.271 (9)	167 (2)
O2*W*—H2*O*2⋯O1*W*	0.83 (1)	1.92 (1)	2.7468 (19)	177 (2)
O3*W*—H1*O*3⋯Cl5^iii^	0.84 (1)	2.41 (1)	3.2139 (13)	159 (2)
O3*W*—H2*O*3⋯Cl2*A* ^iii^	0.84 (1)	2.38 (1)	3.2153 (17)	175 (2)
O3*W*—H2*O*3⋯Cl3*B* ^iii^	0.84 (1)	2.23 (2)	3.05 (2)	167 (2)

Symmetry codes: (i) 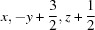; (ii) 
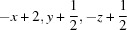
; (iii) 
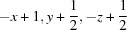
; (iv) 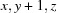.

**Table 2:** Experimental details

Crystal data	
Chemical formula	[Cr(C_6_H_14_N_2_)_3_][ZnCl_4_]Cl·3H_2_O
*M*_r_	691.24
Crystal system, space group	Monoclinic, *P*2_1_/*c*
Temperature (K)	100
*a*, *b*, *c* (Å)	10.594 (2), 13.075 (3), 22.384 (5)
β (°)	100.87 (3)
*V* (Å^3^)	3045.0 (11)
*Z*	4
Radiation type	Synchrotron, λ = 0.62998 Å
μ (mm^−1^)	1.15
Crystal size (mm)	0.25 × 0.15 × 0.05

Data collection	
Diffractometer	ADSC Q210 CCD area detector
Absorption correction	Empirical (using intensity measurements) (*HKL3000sm *SCALEPACK**; Otwinowski & Minor, 1997 ▸)
*T*_min_, *T*_max_	0.762, 0.945
No. of measured, independent and observed [*I* > 2σ(*I*)] reflections	23113, 8090, 7647
*R*_int_	0.034
(sin θ/λ)_max_ (Å^−1^)	0.696

Refinement	
*R*[*F* ^2^ > 2σ(*F* ^2^)], *wR*(*F* ^2^), *S*	0.027, 0.073, 1.05
No. of reflections	8090
No. of parameters	371
No. of restraints	15
H-atom treatment	H atoms treated by a mixture of independent and constrained refinement
Δρ_max_, Δρ_min_ (e Å^−3^)	1.07, −1.14

Computer programs: *PAL BL2D-SMDC* (Shin *et al.*, 2016 ▸), *HKL3000sm* (Otwinowski & Minor, 1997 ▸), *SHELXT2014* (Sheldrick, 2015*a* ▸), *SHELXL2014* (Sheldrick, 2015*b* ▸), *DIAMOND* (Putz & Brandenburg, 2014 ▸) and *publCIF* (Westrip, 2010 ▸).

## supplementary crystallographic information

### Crystal data

**Table:**

[Cr(C_6_H_14_N_2_)_3_][ZnCl_4_]Cl·3H_2_O	*F*(000) = 1444
*M**_r_* = 691.24	*D*_x_ = 1.508 Mg m^−^^3^
Monoclinic, *P*2_1_/*c*	Synchrotron radiation, λ = 0.62998 Å
*a* = 10.594 (2) Å	Cell parameters from 39315 reflections
*b* = 13.075 (3) Å	θ = 0.4–33.6°
*c* = 22.384 (5) Å	µ = 1.15 mm^−^^1^
β = 100.87 (3)°	*T* = 100 K
*V* = 3045.0 (11) Å^3^	Block, yellow
*Z* = 4	0.25 × 0.15 × 0.05 mm

### Data collection

**Table:**

ADSC Q210 CCD area detector diffractometer	7647 reflections with *I* > 2σ(*I*)
Radiation source: PLSII 2D bending magnet	*R*_int_ = 0.034
ω scan	θ_max_ = 26.0°, θ_min_ = 1.7°
Absorption correction: empirical (using intensity measurements) (*HKL3000sm *SCALEPACK**; Otwinowski & Minor, 1997)	*h* = −14→13
*T*_min_ = 0.762, *T*_max_ = 0.945	*k* = −18→18
23113 measured reflections	*l* = −31→23
8090 independent reflections	

### Refinement

**Table:**

Refinement on *F*^2^	15 restraints
Least-squares matrix: full	Hydrogen site location: mixed
*R*[*F*^2^ > 2σ(*F*^2^)] = 0.027	H atoms treated by a mixture of independent and constrained refinement
*wR*(*F*^2^) = 0.073	*w* = 1/[σ^2^(*F*_o_^2^) + (0.0353*P*)^2^ + 1.582*P*] where *P* = (*F*_o_^2^ + 2*F*_c_^2^)/3
*S* = 1.05	(Δ/σ)_max_ = 0.001
8090 reflections	Δρ_max_ = 1.07 e Å^−^^3^
371 parameters	Δρ_min_ = −1.14 e Å^−^^3^

### Special details

**Table:**

Geometry. All esds (except the esd in the dihedral angle between two l.s. planes) are estimated using the full covariance matrix. The cell esds are taken into account individually in the estimation of esds in distances, angles and torsion angles; correlations between esds in cell parameters are only used when they are defined by crystal symmetry. An approximate (isotropic) treatment of cell esds is used for estimating esds involving l.s. planes.

### Fractional atomic coordinates and isotropic or equivalent isotropic displacement parameters (Å^2^)

**Table:**

	*x*	*y*	*z*	*U*_iso_*/*U*_eq_	Occ. (<1)
Cr1	0.74469 (2)	0.93268 (2)	0.34326 (2)	0.00945 (5)	
N1	0.64213 (11)	0.83929 (8)	0.39272 (5)	0.0148 (2)	
H1A	0.6540	0.8610	0.4320	0.018*	
H1B	0.5567	0.8427	0.3766	0.018*	
N2	0.86651 (10)	0.80619 (8)	0.35251 (5)	0.01418 (19)	
H2A	0.8570	0.7719	0.3166	0.017*	
H2B	0.9497	0.8270	0.3629	0.017*	
N3	0.64039 (10)	0.87915 (8)	0.26146 (5)	0.01276 (18)	
H3A	0.6636	0.8136	0.2552	0.015*	
H3B	0.5550	0.8799	0.2625	0.015*	
N4	0.84308 (11)	1.01181 (8)	0.28504 (5)	0.01452 (19)	
H4A	0.8202	1.0790	0.2836	0.017*	
H4B	0.9293	1.0076	0.2991	0.017*	
N5	0.61717 (10)	1.05117 (7)	0.35045 (5)	0.01305 (18)	
H5A	0.6204	1.0989	0.3212	0.016*	
H5B	0.5355	1.0265	0.3453	0.016*	
N6	0.85476 (11)	1.00563 (8)	0.41871 (5)	0.0163 (2)	
H6A	0.8540	0.9680	0.4529	0.020*	
H6B	0.9376	1.0115	0.4135	0.020*	
C1	0.68832 (12)	0.73168 (8)	0.39046 (6)	0.0125 (2)	
H1	0.6558	0.7035	0.3489	0.015*	
C2	0.64271 (14)	0.66233 (9)	0.43714 (6)	0.0179 (2)	
H2C	0.5480	0.6558	0.4273	0.021*	
H2D	0.6665	0.6929	0.4782	0.021*	
C3	0.70450 (15)	0.55615 (9)	0.43674 (6)	0.0208 (3)	
H3C	0.6773	0.5126	0.4682	0.025*	
H3D	0.6748	0.5234	0.3967	0.025*	
C4	0.85043 (15)	0.56437 (10)	0.44909 (6)	0.0220 (3)	
H4C	0.8885	0.4953	0.4484	0.026*	
H4D	0.8806	0.5941	0.4900	0.026*	
C5	0.89466 (13)	0.63175 (9)	0.40110 (6)	0.0175 (2)	
H5C	0.9895	0.6380	0.4102	0.021*	
H5D	0.8694	0.5998	0.3605	0.021*	
C6	0.83391 (12)	0.73752 (9)	0.40071 (5)	0.0126 (2)	
H6	0.8654	0.7702	0.4412	0.015*	
C7	0.66650 (12)	0.94593 (8)	0.21109 (5)	0.0120 (2)	
H7	0.6215	1.0126	0.2134	0.014*	
C8	0.61868 (13)	0.89995 (10)	0.14856 (6)	0.0169 (2)	
H8A	0.5241	0.8921	0.1417	0.020*	
H8B	0.6570	0.8313	0.1464	0.020*	
C9	0.65520 (14)	0.96868 (11)	0.09937 (6)	0.0221 (3)	
H9A	0.6104	1.0351	0.0992	0.026*	
H9B	0.6273	0.9363	0.0590	0.026*	
C10	0.80021 (14)	0.98656 (11)	0.11056 (6)	0.0208 (3)	
H10A	0.8213	1.0329	0.0789	0.025*	
H10B	0.8446	0.9207	0.1075	0.025*	
C11	0.84846 (13)	1.03355 (9)	0.17347 (6)	0.0162 (2)	
H11A	0.9432	1.0405	0.1805	0.019*	
H11B	0.8110	1.1026	0.1752	0.019*	
C12	0.81033 (11)	0.96599 (9)	0.22289 (5)	0.0123 (2)	
H12	0.8560	0.8990	0.2232	0.015*	
C13	0.65454 (12)	1.09834 (9)	0.41220 (6)	0.0133 (2)	
H13	0.6297	1.0503	0.4428	0.016*	
C14	0.58832 (14)	1.20051 (9)	0.41731 (7)	0.0199 (2)	
H14A	0.4940	1.1905	0.4100	0.024*	
H14B	0.6084	1.2482	0.3861	0.024*	
C15	0.63376 (16)	1.24644 (10)	0.48076 (7)	0.0253 (3)	
H15A	0.5931	1.3142	0.4829	0.030*	
H15B	0.6064	1.2016	0.5116	0.030*	
C16	0.77959 (17)	1.25828 (12)	0.49508 (8)	0.0303 (3)	
H16A	0.8063	1.2837	0.5372	0.036*	
H16B	0.8060	1.3094	0.4672	0.036*	
C17	0.84742 (15)	1.15638 (11)	0.48828 (7)	0.0255 (3)	
H17A	0.9413	1.1678	0.4942	0.031*	
H17B	0.8309	1.1081	0.5200	0.031*	
C18	0.79971 (13)	1.10982 (9)	0.42553 (6)	0.0164 (2)	
H18	0.8250	1.1561	0.3942	0.020*	
Zn1A	0.76131 (2)	0.36294 (2)	0.25952 (2)	0.01355 (6)	0.94
Cl1A	0.63241 (4)	0.50140 (3)	0.26301 (2)	0.01450 (9)	0.94
Cl2A	0.86823 (7)	0.38639 (5)	0.18121 (3)	0.02463 (12)	0.94
Cl3A	0.62548 (10)	0.22658 (7)	0.23764 (4)	0.01813 (15)	0.94
Cl4A	0.89500 (4)	0.34751 (3)	0.34937 (2)	0.02371 (8)	0.94
Zn1B	0.7716 (6)	0.3345 (4)	0.2463 (3)	0.0287 (10)	0.06
Cl1B	0.9326 (9)	0.2641 (8)	0.3181 (5)	0.056 (3)	0.06
Cl2B	0.6709 (11)	0.4742 (7)	0.2722 (6)	0.040 (2)	0.06
Cl3B	0.8504 (18)	0.3625 (15)	0.1645 (9)	0.062 (5)	0.06
Cl4B	0.6062 (18)	0.2271 (16)	0.2239 (8)	0.031 (4)	0.06
Cl5	0.76791 (6)	0.60816 (3)	0.03343 (2)	0.04180 (13)	
O1W	0.94431 (12)	0.64959 (9)	0.15743 (6)	0.0304 (3)	
H1O1	0.8960 (19)	0.6389 (15)	0.1230 (6)	0.037*	
H2O1	0.982 (2)	0.7057 (11)	0.1558 (10)	0.037*	
O2W	0.78942 (13)	0.69376 (8)	0.23958 (5)	0.0276 (2)	
H1O2	0.7480 (18)	0.6407 (11)	0.2436 (10)	0.033*	
H2O2	0.8389 (18)	0.6809 (15)	0.2159 (9)	0.033*	
O3W	0.40117 (10)	0.95853 (9)	0.39920 (5)	0.0259 (2)	
H1O3	0.3764 (19)	1.0020 (13)	0.4223 (8)	0.031*	
H2O3	0.3341 (14)	0.9374 (15)	0.3769 (8)	0.031*	

### Atomic displacement parameters (Å^2^)

**Table:**

	*U*^11^	*U*^22^	*U*^33^	*U*^12^	*U*^13^	*U*^23^
Cr1	0.00941 (9)	0.00891 (8)	0.01117 (9)	−0.00020 (6)	0.00483 (7)	0.00071 (6)
N1	0.0136 (5)	0.0139 (4)	0.0196 (5)	0.0054 (3)	0.0102 (4)	0.0066 (4)
N2	0.0111 (5)	0.0135 (4)	0.0204 (5)	0.0001 (3)	0.0094 (4)	0.0001 (4)
N3	0.0125 (5)	0.0125 (4)	0.0143 (5)	−0.0047 (3)	0.0050 (4)	0.0004 (3)
N4	0.0151 (5)	0.0154 (4)	0.0142 (5)	−0.0072 (4)	0.0057 (4)	−0.0016 (4)
N5	0.0126 (5)	0.0117 (4)	0.0145 (5)	0.0015 (3)	0.0019 (4)	0.0012 (3)
N6	0.0112 (5)	0.0196 (5)	0.0178 (5)	0.0035 (4)	0.0023 (4)	−0.0034 (4)
C1	0.0119 (5)	0.0119 (5)	0.0154 (5)	0.0019 (4)	0.0066 (4)	0.0035 (4)
C2	0.0235 (7)	0.0145 (5)	0.0191 (6)	0.0009 (4)	0.0124 (5)	0.0052 (4)
C3	0.0355 (8)	0.0122 (5)	0.0169 (6)	0.0012 (5)	0.0109 (5)	0.0025 (4)
C4	0.0328 (8)	0.0146 (5)	0.0184 (6)	0.0089 (5)	0.0042 (5)	0.0027 (4)
C5	0.0185 (6)	0.0141 (5)	0.0205 (6)	0.0070 (4)	0.0053 (5)	−0.0009 (4)
C6	0.0118 (5)	0.0117 (5)	0.0152 (5)	0.0026 (4)	0.0045 (4)	0.0004 (4)
C7	0.0127 (5)	0.0122 (4)	0.0121 (5)	−0.0024 (4)	0.0050 (4)	0.0008 (4)
C8	0.0162 (6)	0.0203 (6)	0.0143 (5)	−0.0045 (4)	0.0033 (4)	−0.0025 (4)
C9	0.0229 (7)	0.0294 (7)	0.0139 (6)	−0.0025 (5)	0.0038 (5)	0.0017 (5)
C10	0.0225 (7)	0.0270 (6)	0.0149 (6)	−0.0026 (5)	0.0089 (5)	0.0008 (5)
C11	0.0170 (6)	0.0176 (5)	0.0162 (5)	−0.0049 (4)	0.0088 (4)	0.0019 (4)
C12	0.0127 (5)	0.0126 (5)	0.0130 (5)	−0.0030 (4)	0.0058 (4)	−0.0004 (4)
C13	0.0140 (5)	0.0108 (5)	0.0156 (5)	0.0015 (4)	0.0041 (4)	−0.0001 (4)
C14	0.0227 (6)	0.0123 (5)	0.0265 (7)	0.0055 (4)	0.0096 (5)	0.0000 (4)
C15	0.0329 (8)	0.0169 (6)	0.0294 (7)	0.0023 (5)	0.0145 (6)	−0.0063 (5)
C16	0.0357 (9)	0.0223 (6)	0.0345 (8)	−0.0063 (6)	0.0104 (7)	−0.0146 (6)
C17	0.0231 (7)	0.0269 (7)	0.0256 (7)	−0.0030 (5)	0.0020 (6)	−0.0132 (5)
C18	0.0149 (6)	0.0145 (5)	0.0202 (6)	−0.0010 (4)	0.0047 (4)	−0.0048 (4)
Zn1A	0.00992 (9)	0.01085 (11)	0.02095 (13)	0.00062 (7)	0.00562 (8)	0.00178 (7)
Cl1A	0.01152 (18)	0.01180 (19)	0.02058 (16)	0.00198 (12)	0.00407 (13)	−0.00062 (13)
Cl2A	0.0129 (2)	0.0329 (3)	0.0317 (3)	0.00436 (17)	0.0133 (2)	0.0052 (2)
Cl3A	0.0171 (4)	0.01019 (19)	0.0277 (4)	−0.0013 (2)	0.0058 (3)	−0.0002 (2)
Cl4A	0.02115 (17)	0.01976 (15)	0.02746 (18)	0.00188 (11)	−0.00247 (13)	0.00251 (12)
Zn1B	0.029 (2)	0.027 (2)	0.034 (3)	−0.0094 (19)	0.0149 (16)	−0.0010 (17)
Cl1B	0.037 (4)	0.069 (6)	0.059 (6)	−0.013 (4)	−0.002 (4)	0.028 (5)
Cl2B	0.037 (5)	0.019 (4)	0.071 (7)	−0.017 (3)	0.030 (5)	−0.020 (4)
Cl3B	0.041 (9)	0.085 (12)	0.066 (11)	0.026 (7)	0.028 (8)	−0.001 (7)
Cl4B	0.010 (4)	0.034 (5)	0.045 (8)	0.010 (3)	−0.008 (4)	−0.004 (5)
Cl5	0.0815 (4)	0.03036 (19)	0.01572 (16)	−0.0234 (2)	0.01466 (19)	−0.00282 (13)
O1W	0.0211 (5)	0.0298 (5)	0.0381 (7)	−0.0013 (4)	0.0000 (5)	0.0111 (5)
O2W	0.0423 (7)	0.0146 (4)	0.0300 (6)	−0.0058 (4)	0.0168 (5)	−0.0031 (4)
O3W	0.0156 (5)	0.0347 (6)	0.0285 (5)	0.0001 (4)	0.0071 (4)	−0.0024 (4)

### Geometric parameters (Å, º)

**Table:**

Cr1—N3	2.0737 (12)	C8—H8A	0.9900
Cr1—N5	2.0817 (10)	C8—H8B	0.9900
Cr1—N2	2.0839 (11)	C9—C10	1.527 (2)
Cr1—N1	2.0859 (11)	C9—H9A	0.9900
Cr1—N4	2.0899 (11)	C9—H9B	0.9900
Cr1—N6	2.0928 (12)	C10—C11	1.5330 (19)
N1—C1	1.4937 (15)	C10—H10A	0.9900
N1—H1A	0.9100	C10—H10B	0.9900
N1—H1B	0.9100	C11—C12	1.5283 (16)
N2—C6	1.4932 (15)	C11—H11A	0.9900
N2—H2A	0.9100	C11—H11B	0.9900
N2—H2B	0.9100	C12—H12	1.0000
N3—C7	1.4927 (15)	C13—C18	1.5178 (18)
N3—H3A	0.9100	C13—C14	1.5232 (16)
N3—H3B	0.9100	C13—H13	1.0000
N4—C12	1.4942 (15)	C14—C15	1.533 (2)
N4—H4A	0.9100	C14—H14A	0.9900
N4—H4B	0.9100	C14—H14B	0.9900
N5—C13	1.4971 (16)	C15—C16	1.525 (2)
N5—H5A	0.9100	C15—H15A	0.9900
N5—H5B	0.9100	C15—H15B	0.9900
N6—C18	1.5009 (16)	C16—C17	1.535 (2)
N6—H6A	0.9100	C16—H16A	0.9900
N6—H6B	0.9100	C16—H16B	0.9900
C1—C6	1.5179 (17)	C17—C18	1.5270 (19)
C1—C2	1.5289 (16)	C17—H17A	0.9900
C1—H1	1.0000	C17—H17B	0.9900
C2—C3	1.5356 (18)	C18—H18	1.0000
C2—H2C	0.9900	Zn1A—Cl4A	2.2400 (9)
C2—H2D	0.9900	Zn1A—Cl1A	2.2779 (6)
C3—C4	1.522 (2)	Zn1A—Cl2A	2.2799 (9)
C3—H3C	0.9900	Zn1A—Cl3A	2.2856 (10)
C3—H3D	0.9900	Zn1B—Cl3B	2.18 (2)
C4—C5	1.529 (2)	Zn1B—Cl4B	2.23 (2)
C4—H4C	0.9900	Zn1B—Cl2B	2.246 (11)
C4—H4D	0.9900	Zn1B—Cl1B	2.304 (11)
C5—C6	1.5247 (16)	Cl1B—O1W^i^	1.993 (11)
C5—H5C	0.9900	O1W—Cl1B^ii^	1.993 (11)
C5—H5D	0.9900	O1W—H1O1	0.853 (9)
C6—H6	1.0000	O1W—H2O1	0.841 (9)
C7—C12	1.5194 (17)	O2W—H1O2	0.834 (9)
C7—C8	1.5197 (17)	O2W—H2O2	0.829 (9)
C7—H7	1.0000	O3W—H1O3	0.843 (9)
C8—C9	1.5267 (19)	O3W—H2O3	0.835 (9)
			
N3—Cr1—N5	94.24 (5)	C12—C7—C8	112.19 (10)
N3—Cr1—N2	92.11 (5)	N3—C7—H7	108.2
N5—Cr1—N2	169.03 (4)	C12—C7—H7	108.2
N3—Cr1—N1	91.55 (5)	C8—C7—H7	108.2
N5—Cr1—N1	89.09 (4)	C7—C8—C9	110.21 (10)
N2—Cr1—N1	81.81 (4)	C7—C8—H8A	109.6
N3—Cr1—N4	82.06 (4)	C9—C8—H8A	109.6
N5—Cr1—N4	94.99 (5)	C7—C8—H8B	109.6
N2—Cr1—N4	94.74 (5)	C9—C8—H8B	109.6
N1—Cr1—N4	172.64 (4)	H8A—C8—H8B	108.1
N3—Cr1—N6	171.68 (4)	C8—C9—C10	110.83 (12)
N5—Cr1—N6	82.49 (5)	C8—C9—H9A	109.5
N2—Cr1—N6	92.35 (5)	C10—C9—H9A	109.5
N1—Cr1—N6	96.04 (5)	C8—C9—H9B	109.5
N4—Cr1—N6	90.58 (5)	C10—C9—H9B	109.5
C1—N1—Cr1	109.07 (7)	H9A—C9—H9B	108.1
C1—N1—H1A	109.9	C9—C10—C11	111.28 (11)
Cr1—N1—H1A	109.9	C9—C10—H10A	109.4
C1—N1—H1B	109.9	C11—C10—H10A	109.4
Cr1—N1—H1B	109.9	C9—C10—H10B	109.4
H1A—N1—H1B	108.3	C11—C10—H10B	109.4
C6—N2—Cr1	109.01 (7)	H10A—C10—H10B	108.0
C6—N2—H2A	109.9	C12—C11—C10	110.21 (10)
Cr1—N2—H2A	109.9	C12—C11—H11A	109.6
C6—N2—H2B	109.9	C10—C11—H11A	109.6
Cr1—N2—H2B	109.9	C12—C11—H11B	109.6
H2A—N2—H2B	108.3	C10—C11—H11B	109.6
C7—N3—Cr1	109.01 (7)	H11A—C11—H11B	108.1
C7—N3—H3A	109.9	N4—C12—C7	106.28 (10)
Cr1—N3—H3A	109.9	N4—C12—C11	113.29 (9)
C7—N3—H3B	109.9	C7—C12—C11	111.54 (10)
Cr1—N3—H3B	109.9	N4—C12—H12	108.5
H3A—N3—H3B	108.3	C7—C12—H12	108.5
C12—N4—Cr1	109.05 (7)	C11—C12—H12	108.5
C12—N4—H4A	109.9	N5—C13—C18	107.70 (10)
Cr1—N4—H4A	109.9	N5—C13—C14	112.66 (10)
C12—N4—H4B	109.9	C18—C13—C14	111.25 (10)
Cr1—N4—H4B	109.9	N5—C13—H13	108.4
H4A—N4—H4B	108.3	C18—C13—H13	108.4
C13—N5—Cr1	108.39 (7)	C14—C13—H13	108.4
C13—N5—H5A	110.0	C13—C14—C15	110.15 (11)
Cr1—N5—H5A	110.0	C13—C14—H14A	109.6
C13—N5—H5B	110.0	C15—C14—H14A	109.6
Cr1—N5—H5B	110.0	C13—C14—H14B	109.6
H5A—N5—H5B	108.4	C15—C14—H14B	109.6
C18—N6—Cr1	109.12 (8)	H14A—C14—H14B	108.1
C18—N6—H6A	109.9	C16—C15—C14	111.29 (12)
Cr1—N6—H6A	109.9	C16—C15—H15A	109.4
C18—N6—H6B	109.9	C14—C15—H15A	109.4
Cr1—N6—H6B	109.9	C16—C15—H15B	109.4
H6A—N6—H6B	108.3	C14—C15—H15B	109.4
N1—C1—C6	106.11 (9)	H15A—C15—H15B	108.0
N1—C1—C2	112.82 (10)	C15—C16—C17	111.45 (12)
C6—C1—C2	111.69 (10)	C15—C16—H16A	109.3
N1—C1—H1	108.7	C17—C16—H16A	109.3
C6—C1—H1	108.7	C15—C16—H16B	109.3
C2—C1—H1	108.7	C17—C16—H16B	109.3
C1—C2—C3	110.02 (10)	H16A—C16—H16B	108.0
C1—C2—H2C	109.7	C18—C17—C16	110.94 (13)
C3—C2—H2C	109.7	C18—C17—H17A	109.5
C1—C2—H2D	109.7	C16—C17—H17A	109.5
C3—C2—H2D	109.7	C18—C17—H17B	109.5
H2C—C2—H2D	108.2	C16—C17—H17B	109.5
C4—C3—C2	110.76 (11)	H17A—C17—H17B	108.0
C4—C3—H3C	109.5	N6—C18—C13	106.80 (10)
C2—C3—H3C	109.5	N6—C18—C17	112.82 (11)
C4—C3—H3D	109.5	C13—C18—C17	111.57 (11)
C2—C3—H3D	109.5	N6—C18—H18	108.5
H3C—C3—H3D	108.1	C13—C18—H18	108.5
C3—C4—C5	110.38 (11)	C17—C18—H18	108.5
C3—C4—H4C	109.6	Cl4A—Zn1A—Cl1A	108.81 (2)
C5—C4—H4C	109.6	Cl4A—Zn1A—Cl2A	112.38 (3)
C3—C4—H4D	109.6	Cl1A—Zn1A—Cl2A	107.91 (3)
C5—C4—H4D	109.6	Cl4A—Zn1A—Cl3A	112.84 (3)
H4C—C4—H4D	108.1	Cl1A—Zn1A—Cl3A	105.66 (4)
C6—C5—C4	109.98 (11)	Cl2A—Zn1A—Cl3A	108.92 (3)
C6—C5—H5C	109.7	Cl3B—Zn1B—Cl4B	109.0 (8)
C4—C5—H5C	109.7	Cl3B—Zn1B—Cl2B	110.8 (6)
C6—C5—H5D	109.7	Cl4B—Zn1B—Cl2B	100.3 (7)
C4—C5—H5D	109.7	Cl3B—Zn1B—Cl1B	107.6 (6)
H5C—C5—H5D	108.2	Cl4B—Zn1B—Cl1B	110.7 (6)
N2—C6—C1	106.74 (10)	Cl2B—Zn1B—Cl1B	118.1 (5)
N2—C6—C5	113.24 (10)	O1W^i^—Cl1B—Zn1B	148.4 (7)
C1—C6—C5	111.78 (10)	Cl1B^ii^—O1W—H1O1	128.5 (15)
N2—C6—H6	108.3	Cl1B^ii^—O1W—H2O1	20.4 (15)
C1—C6—H6	108.3	H1O1—O1W—H2O1	108.4 (17)
C5—C6—H6	108.3	H1O2—O2W—H2O2	108.0 (17)
N3—C7—C12	106.99 (10)	H1O3—O3W—H2O3	105.2 (17)
N3—C7—C8	112.78 (9)		
			
Cr1—N1—C1—C6	43.74 (11)	Cr1—N4—C12—C11	165.21 (8)
Cr1—N1—C1—C2	166.35 (9)	N3—C7—C12—N4	−56.47 (11)
N1—C1—C2—C3	−174.32 (11)	C8—C7—C12—N4	179.35 (9)
C6—C1—C2—C3	−54.88 (14)	N3—C7—C12—C11	179.60 (9)
C1—C2—C3—C4	56.88 (15)	C8—C7—C12—C11	55.42 (13)
C2—C3—C4—C5	−58.90 (14)	C10—C11—C12—N4	−174.49 (11)
C3—C4—C5—C6	57.83 (14)	C10—C11—C12—C7	−54.61 (14)
Cr1—N2—C6—C1	43.07 (10)	Cr1—N5—C13—C18	43.53 (10)
Cr1—N2—C6—C5	166.49 (8)	Cr1—N5—C13—C14	166.59 (9)
N1—C1—C6—N2	−57.08 (12)	N5—C13—C14—C15	−178.16 (11)
C2—C1—C6—N2	179.59 (10)	C18—C13—C14—C15	−57.11 (15)
N1—C1—C6—C5	178.59 (10)	C13—C14—C15—C16	56.49 (16)
C2—C1—C6—C5	55.27 (13)	C14—C15—C16—C17	−55.32 (18)
C4—C5—C6—N2	−176.70 (11)	C15—C16—C17—C18	54.00 (18)
C4—C5—C6—C1	−56.10 (14)	Cr1—N6—C18—C13	40.46 (11)
Cr1—N3—C7—C12	43.41 (10)	Cr1—N6—C18—C17	163.39 (10)
Cr1—N3—C7—C8	167.23 (8)	N5—C13—C18—N6	−55.59 (12)
N3—C7—C8—C9	−176.66 (11)	C14—C13—C18—N6	−179.51 (10)
C12—C7—C8—C9	−55.76 (14)	N5—C13—C18—C17	−179.30 (10)
C7—C8—C9—C10	56.43 (15)	C14—C13—C18—C17	56.79 (14)
C8—C9—C10—C11	−57.35 (15)	C16—C17—C18—N6	−174.93 (12)
C9—C10—C11—C12	55.91 (15)	C16—C17—C18—C13	−54.69 (16)
Cr1—N4—C12—C7	42.38 (10)		

Symmetry codes: (i) −*x*+2, *y*−1/2, −*z*+1/2; (ii) −*x*+2, *y*+1/2, −*z*+1/2.

### Hydrogen-bond geometry (Å, º)

**Table:**

*D*—H···*A*	*D*—H	H···*A*	*D*···*A*	*D*—H···*A*
N1—H1*A*···Cl5^iii^	0.91	2.40	3.2535 (15)	157
N1—H1*B*···O3*W*	0.91	2.36	3.0178 (16)	129
N2—H2*A*···O2*W*	0.91	2.01	2.9051 (17)	166
N2—H2*B*···Cl2*A*^ii^	0.91	2.45	3.2197 (14)	142
N2—H2*B*···Cl3*B*^ii^	0.91	2.36	3.180 (18)	150
N3—H3*A*···O2*W*	0.91	2.13	2.9832 (16)	156
N3—H3*B*···Cl1*A*^iv^	0.91	2.52	3.2574 (13)	138
N3—H3*B*···Cl3*A*^iv^	0.91	2.77	3.4547 (16)	133
N3—H3*B*···Cl2*B*^iv^	0.91	2.67	3.471 (10)	147
N3—H3*B*···Cl4*B*^iv^	0.91	2.68	3.35 (2)	131
N4—H4*A*···Cl1*B*^v^	0.91	2.74	3.473 (11)	138
N4—H4*B*···Cl2*A*^ii^	0.91	2.64	3.4267 (15)	146
N4—H4*B*···O1*W*^ii^	0.91	2.39	2.9804 (17)	123
N5—H5*A*···Cl3*A*^v^	0.91	2.51	3.4245 (14)	178
N5—H5*A*···Cl4*B*^v^	0.91	2.73	3.634 (19)	173
N5—H5*B*···Cl1*A*^iv^	0.91	2.74	3.3664 (16)	127
N5—H5*B*···O3*W*	0.91	2.22	2.9724 (17)	140
N6—H6*A*···Cl5^iii^	0.91	2.39	3.2474 (14)	158
O1*W*—H1*O*1···Cl5	0.85 (1)	2.24 (1)	3.0878 (17)	179 (2)
O1*W*—H2*O*1···Cl4*A*^ii^	0.84 (1)	2.28 (1)	3.1170 (13)	174 (2)
O2*W*—H1*O*2···Cl1*A*	0.83 (1)	2.28 (1)	3.1140 (12)	175 (2)
O2*W*—H1*O*2···Cl2*B*	0.83 (1)	2.45 (1)	3.271 (9)	167 (2)
O2*W*—H2*O*2···O1*W*	0.83 (1)	1.92 (1)	2.7468 (19)	177 (2)
O3*W*—H1*O*3···Cl5^iv^	0.84 (1)	2.41 (1)	3.2139 (13)	159 (2)
O3*W*—H2*O*3···Cl2*A*^iv^	0.84 (1)	2.38 (1)	3.2153 (17)	175 (2)
O3*W*—H2*O*3···Cl3*B*^iv^	0.84 (1)	2.23 (2)	3.05 (2)	167 (2)

Symmetry codes: (ii) −*x*+2, *y*+1/2, −*z*+1/2; (iii) *x*, −*y*+3/2, *z*+1/2; (iv) −*x*+1, *y*+1/2, −*z*+1/2; (v) *x*, *y*+1, *z*.
